# A dual autoencoder and singular value decomposition based feature optimization for the segmentation of brain tumor from MRI images

**DOI:** 10.1186/s12880-021-00614-3

**Published:** 2021-05-13

**Authors:** K. Aswani, D. Menaka

**Affiliations:** 1 Noorul Islam Centre for Higher Education, Kumrancoil, Tamil Nadu India; 2 Department of Applied Electronics, Noorul Islam Center for Higher Education, Kumrancoil, Tamil Nadu India; 3 Malappuram, India

**Keywords:** MRI, Brain tumor, Deep learning, Computer vision, Anomaly prediction

## Abstract

**Background:**

The brain tumor is the growth of abnormal cells inside the brain. These cells can be grown into malignant or benign tumors. Segmentation of tumor from MRI images using image processing techniques started decades back. Image processing based brain tumor segmentation can be divided in to three categories conventional image processing methods, Machine Learning methods and Deep Learning methods. Conventional methods lacks the accuracy in segmentation due to complex spatial variation of tumor. Machine Learning methods stand as a good alternative to conventional methods. Methods like SVM, KNN, Fuzzy and a combination of either of these provide good accuracy with reasonable processing speed. The difficulty in processing the various feature extraction methods and maintain accuracy as per the medical standards still exist as a limitation for machine learning methods. In Deep Learning features are extracted automatically in various stages of the network and maintain accuracy as per the medical standards. Huge database requirement and high computational time is still poses a problem for deep learning. To overcome the limitations specified above we propose an unsupervised dual autoencoder with latent space optimization here. The model require only normal MRI images for its training thus reducing the huge tumor database requirement. With a set of normal class data, an autoencoder can reproduce the feature vector into an output layer. This trained autoencoder works well with normal data while it fails to reproduce an anomaly to the output layer. But a classical autoencoder suffer due to poor latent space optimization. The Latent space loss of classical autoencoder is reduced using an auxiliary encoder along with the feature optimization based on singular value decomposition (SVD). The patches used for training are not traditional square patches but we took both horizontal and vertical patches to keep both local and global appearance features on the training set. An Autoencoder is applied separately for learning both horizontal and vertical patches. While training a logistic sigmoid transfer function is used for both encoder and decoder parts. SGD optimizer is used for optimization with an initial learning rate of .001 and the maximum epochs used are 4000. The network is trained in MATLAB 2018a with a processor capacity of 3.7 GHz with NVIDIA GPU and 16 GB of RAM.

**Results:**

The results are obtained using a patch size of 16 × 64, 64 × 16 for horizontal and vertical patches respectively. In Glioma images tumor is not grown from a point rather it spreads randomly. Region filling and connectivity operations are performed to get the final tumor segmentation. Overall the method segments Meningioma better than Gliomas. Three evaluation metrics are considered to measure the performance of the proposed system such as Dice Similarity Coefficient, Positive Predictive Value, and Sensitivity.

**Conclusion:**

An unsupervised method for the segmentation of brain tumor from MRI images is proposed here. The proposed dual autoencoder with SVD based feature optimization reduce the latent space loss in the classical autoencoder. The proposed method have advantages in computational efficiency, no need of huge database requirement and better accuracy than machine learning methods. The method is compared Machine Learning methods Like SVM, KNN and supervised deep learning methods like CNN and commentable results are obtained.

## Introduction

The brain tumor is a serious medical condition if not treated earlier will reduce the life span of the affected person. World Health Organization (WHO) classifies the tumor as benign and malignant. In malignant, the tumor has Type-I to Type-IV varieties. Gliomas and Meningioma are malignant tumors that start as Type-I which affects the brain and spinal cord. The affected persons experience strong headaches, seizures, loss of balance, and weight loss. Treatments like surgery, radiation, and chemotherapy are suggested by the medical experts to either completely cure or partially ceases the growth of tumors. It is important to detect the tumor in an early stage to make use of the full effect of these treatments. The doctors perform the MRI scanning to examine the potential growth of the tumor inside the brain. These actions are performed by experts in radiography treatments. Manual inspection of cell growth often leads to judgment error and can be replaced by modern automatic analysis methods. Processing MRI images for detection and segmentation of tumor in the brain is an alternative method which can alleviate the error caused by manual inspection.

Accurate segmentation of Tumor and by understanding its inter-tumoral structure is important for treatment planning and follow up. The physicians use some rough measures for their evaluation and it leads to detection errors. For the reasons specified above a semi-automatic or fully automatic method should be developed for tumor segmentation. Developing an automatic method is a challenging task due to the diversification in tumor pixels, intensity inhomogeneity, noise effects, and difficulty in separating tumor mass from surrounding pixels. Various parametric and non-parametric models were developed previously for tumor detection. Deep learning is a new technology which can lead to accurate segmentation of tumor pixels but the training procedure of the same has to meet the computational cost and large database requirement. Researches are going on in the direction of treating tumor pixels as outliers compared to normal MRI pixels. These methods reduce the computational cost and database required for previous deep learning methods.

## Review of literature

Automatic detection of tumor starts from simple thresholding [1, 2] and developed to various sophisticated methods like Deep Learning (DL). The methods are classified into three categories. At first, we have the segmentation through conventional image processing by several methods. Then Machine Learning (ML) with various feature extraction methods and finally the DL methods. The selection of a particular method is based on the problem selected. In medical diagnostics, the importance is given to accuracy in segmentation rather than the speed of operation. Here DL has a clear advantage over the other methods. The segmentation through conventional image processing is the simple image processing operations on the pixel values of the MRI like multilevel thresholding [3, 4]. Various methods for segmenting the required portion from an MRI is developed over the years. Some of these are Fuzzy Clustering [5], Watershed algorithm [6], Markov Random Field [7], and Genetic algorithm [8]. All the above method has advantages like the speed of operation which is useful if fast results are required. But they lack the performance when there are pixel intensity variations, diverse nature of the tumor, machine, and other noise effects.

To overcome the deficiencies specified above special features are extracted from the MRI belong to the tumor and other parts. These features are then used to train a classifier that can predict the tumor pixel by pixel. The method is generally called Machine Learning. In our study, it is shown that some of the methods which segment the tumor accurately compared to the general image processing techniques. Some of the methods are Support Vector Machine (SVM) [9–12] Random Forest (RF) [13–15] and Naïve Bayesian (NB) [16], K nearest neighbor (KNN) [17–19], Artificial Neural Networks (ANN) [20–23] and hybrid methods [24–26]. All the methods above require special features separating a tumor pixel from a Non-tumor pixel. The accuracy of the methods depends on the feature value and the number of features extracted. Various methods are available for extracting low-level and high-level features from the MRI images. Since the classifiers need to be trained before classification a large number of data is required. The database can be collected from hospitals, or there is an open-source database for tumor detection challenges. One of such databases is BRATS. The database has different versions starting from 2012. Using the same database for different methods makes the comparison study effective. So, most of the methods discussed here use the BRATS 2015 database for training and testing.

Deep Learning does not require the features for training and testing. Features are extracted from the different layers in the training procedure. So, it makes the method user friendly but the complex nature of designing the deep layers emphasize the expertise required in the field. Segmentation of tumors using Convolutional Neural Network (CNN) [27] is popular among tumor detection methods. In [28] authors present a multichannel input CNN for feature extraction using CNN. Instead of giving the patches to the input layer, the authors find the superpixel segmentation of the image first because of saliency detection then each superpixel is applied to a different CNN architecture. Each CNN architecture generates hierarchical features and pooled to combine the final feature set. In [29, 30] the authors presented two experimental CNN architecture for multichannel input namely the Exception model and the Dense Net model. A high feature recognition rate is obtained as claimed by the authors. Like ML a huge database is required for training the network. In [31, 32] the authors present the latest GAN model to segment the brain tumor. In [32] they developed a model named RescueNet which uses unpaired adversarial training to segment the whole tumor followed by core and enhance regions in a brain MRI scan. The authors claim the method can reduce the labeled data needed for deep learning. A 3D volume-to-volume GAN is developed in [33] for the segmentation 3D MRI images. BRATs 2015 dataset is used for testing and the authors claim good Dice score for their model. In [34] the authors present a survey on deep learning based brain tumor segmentation. The different models used in deep learning were CNN, FCN, Cascaded CNN, RNN, Generative model and Ensemble models. In [35], the authors present an Autoencoder-based anomaly prediction. Different type of Variational Auto Encoder (VAE) is implemented and their performance is measured. The reconstruction is not the best due to the latent space loss and poor optimization. The training process in CNN and Generative Adversarial Network (GAN) [31–34] is both complex and time-consuming. So, in this paper, we suggest a simple autoencoder based training. Instead of training on tumor pixels, autoencoder training is performed on normal pixels. The tumor is detected as an anomaly present in the brain. We hope this work will reduce the complexity and huge data requirement required for CNN and other supervised deep learning methods.

To overcome the limitations specified above here we propose a Dual Autoencoder-based anomaly prediction for brain tumor detection. The main contribution of our work is as follows.

1. Instead of conventional square patches, we employ both horizontal and vertical patches for training an Autoencoder for normal brain image detection. This helps to keep more details inside the patch because of the heterogeneous nature of tumors in MRI images.
2. We employed separate Autoencoder for horizontal and vertical patches. An auxiliary encoder is also used to obtain useful latent space features. singular value decomposition (SVD) based feature optimization is performed further.
3. The performance of the proposed method is analyzed and compared with existing Autoencoder-based anomaly predictors as well as deep learning and machine learning methods.

## Methodology

### Method

**Fig. 1:**
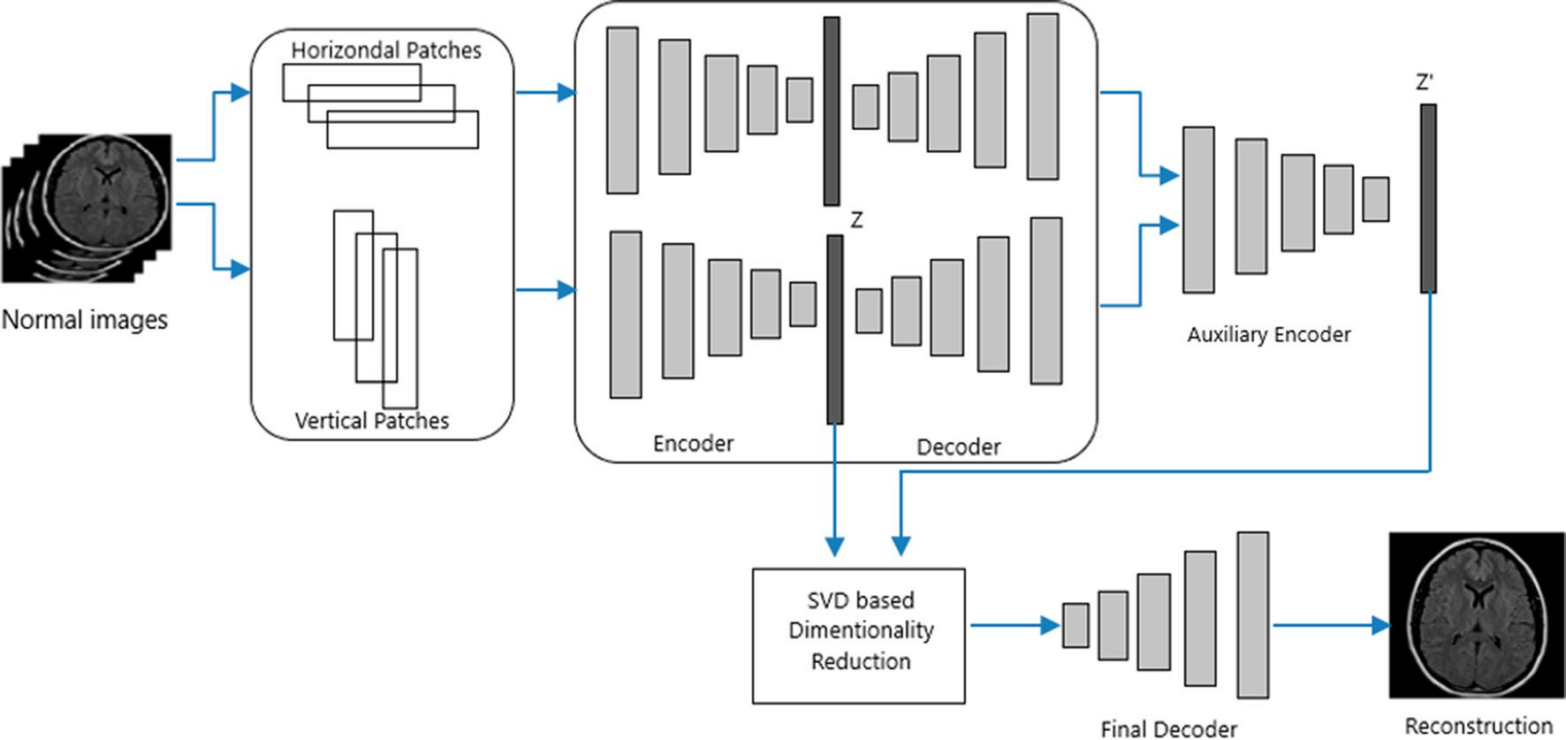
Overview of the proposed method

The architecture of the proposed method is presented in Fig. 1. There are four stages in the overall approach. At first, we extract the horizontal and vertical patches from the training set. Two autoencoders are designed for training the patches separately. Latent space information from two primary encoders named as Z and Z' from an auxiliary encoder are combined for dimensionality reduction using singular value decomposition (SVD). Finally, the optimized features are fed to a decoder for the reconstruction of normal MRI images.

### Patch extraction

Instead of traditional square patches we used horizontal and vertical patches. This will keep global and local appearance features in the patches. A dimension of 16 × 64 and 64 × 16 is kept for horizontal and vertical patches respectively. The mean intensity and variance of all the patches in the training set are extracted. All the patches are then normalized with zero mean and unit variance before fed into the autoencoder for training.

### Autoencoder architecture

A classical autoencoder architecture is used here. The encoder network $Encode_{\theta }\left( X \right)$ with θ as the parameter project the training samples $X$ to a lower-dimensional space called $Z$. Here the design consist of two autoencoders both for horizontal and vertical patches. The $Decode_{\Phi } \left( X \right)$ function then try to reconstruct the original samples from the latent space representation $Z$. Hence the network tries to recreate the normal brain samples from its lower dimensions by minimizing the loss function.

1 $$L_{autoenc} = \left( {X^{c} - \hat{X}^{c} } \right)$$

where $X^{c}$ is a horizontal or vertical patch and $\hat{X}^{c}$ is the patch reconstructed by the autoencoder. Here the autoencoder tries to reduce the $l1$ distance between the input patch and the reconstructed patch in both horizontal and vertical directions. The idea is to minimize the loss between input and output samples so that the network fails to identify the anomaly samples present in the brain MRI. $Z$ is called the *latent space* or *manifold* representation of the input patches, which is either represented in the form of a 1-D vector or a higher-order vector in case of high-resolution MRI images for keeping the spatial context data for generating the high-quality data while reconstruction.

### Auxiliary encoder

An auxiliary encoder is adopted here for reducing the latent feature distance between the horizontal and vertical patches. While reconstructing the patches from both directions the decoders try to keep as much spatial information as possible. This creates a bottleneck while reconstructing the final image from patches. The auxiliary encoder reduces the difference in the latent space features of both horizontal and vertical encoders. One of the problems faced by classical autoencoder is the distribution distortion in the latent space. This also can be reduced using the latest features of auxiliary encoder $Z^{\prime}$ as a supporting feature for $Z$. Both the encoders in the first stage and auxiliary encoder has latent loss $L_{en}$ s and $L_{aux\_enc}$ respectively.

### Lower rank representation

SVD is a method used to represent a higher-order matrix to lower-order models. This is widely used as a discriminative model for outlier detection. The lower-dimensional data $Z$ from the dual autoencoder along with the auxiliary encoder $Z^{\prime}$ should be constrained. The variation image for each patch is localized so that it can be mapped to a lower-dimensional space. For each patch, there is a lower-dimensional latent representation $z_{1} ,z_{2} \ldots z_{n}$. To construct a lower rank representation we optimize the following constraints.

2 $$min _{{z_{1} ,z_{2} \ldots z_{n} }} \mathop \sum \limits_{n = 1}^{T} y_{l} - M_{n} Z_{n2}^{2} ;st. rank\left( Z \right) = r$$

where $y_{l} ,M_{n}$ represents the measurement sequence and measurement matrix respectively.

### Image reconstruction

The lower-dimensional data-optimized using SVD is fed to the final decoder stage for the proper reconstruction of the images. If the input to an autoencoder is $x \in {\mathbb{R}}^{{D_{x} }}$, then the encoder maps the vector $x$ into a vector $z \in {\mathbb{R}}^{{D^{\left( 1 \right)} }}$ as follows:

3 $$z^{\left( 1 \right)} = h^{\left( 1 \right)} \left( {W^{\left( 1 \right)} x + b^{\left( 1 \right)} } \right)$$

where the superscript (1) indicates the first layer and $h^{\left( 1 \right)} :{\mathbb{R}}^{{D^{\left( 1 \right)} }} \to {\mathbb{R}}^{{D^{\left( 1 \right)} }}$ is the transfer function for the encoder, $W^{\left( 1 \right)} \in {\mathbb{R}}^{{D^{\left( 1 \right)} \times D_{x} }}$ is the weight matrix and $b^{\left( 1 \right)} \in {\mathbb{R}}^{{D^{\left( 1 \right)} }}$ is the bias vector.

The decoder maps the encoded data $z$ into an approximation of $x$ as follows:

4 $$\hat{x} = h^{\left( 2 \right)} \left( {W^{\left( 2 \right)} z + b^{\left( 2 \right)} } \right)$$

where the superscript (2) represents the second layer. $h^{\left( 2 \right)} :{\mathbb{R}}^{{D_{x} }} \to {\mathbb{R}}^{{D_{x} }}$ is the transfer function for the decoder, $W^{\left( 2 \right)} \in {\mathbb{R}}^{{ D_{x} \times D^{\left( 1 \right)} }}$ is the weight matrix and $b^{\left( 2 \right)} \in {\mathbb{R}}^{{D_{x} }}$ is the bias vector. The image reconstruction loss while minimizing the distance between the input vector and output vector during this process is as follows:

5 $${L_{rec}} = {{\mathbb{E}}_{x \sim {P_x}}}{\left\| {x - {x^ \wedge }} \right\|_1}$$

So that the overall loss function is the sum of 2 encoders, 2 decoder loss along with auxiliary encoder, the low-rank loss, and the reconstruction loss. It is represented as follows:

6 $$L_{TOT} = L_{Enc1} + L_{Enc2} + L_{aux\_rec1} + L_{aux\_rec1} + L_{aux\_enc} + L_{SVD} + L_{rec}$$

### Segmentation procedure

The dual autoencoder architecture explained above is able to reproduce the ‘normal’ MRI pixels successfully because this is what it trained to do. The total loss $L_{TOT}$ is also minimized in this case. But for a tumor image it failed to reconstruct the image effectively. The enhanced loss during tumor image reconstruction represents the failed detection of tumor pixels. We use some morphological image processing methods to segment the tumor portions from non tumor parts. Both the input and reproduced images are converted to binary format using average grey level threshold of both. Then binary image subtraction is performed to find the possible differences between the input and reproduced images. Some small regions is erroneously detected as tumor portions. To deal with that we impose area based constraints for filtering out region areas less than a predefined threshold.

The experimental procedure is describes as follows.

A. **Database**
  Four sets of data each consist of 760 images are considered for training and evaluation. A single image is having a size of 512 × 512. There are 3 namely T1-Weighted, T2-Weighted, FLAIR images are available in an MRI dataset. Our dataset contains T1-weighted contrast enhanced images. The database has Low-Grade Gliomas (LGG), High-Grade Gliomas (HGG), meningioma, anaplastic astrocytoma, and glioblastoma multiform tumor images. Since our work mainly focuses on detecting gliomas and meningioma the images corresponding to this area selected from the database. Normal MRI images are collected from the HCP dataset. All the datasets were normalized to maintain the uniformity before going to patch extraction. All the images are then aligned and the skull is stripped before the training setup. The patches are extracted from normal images as well as non-tumor part of the tumor images.
B. **Setup**
  Normal MRI images are employed in the training process. The MRI images are prone to noise due to moving parts in the scanner and various electronic components noise. The noise effect is not treated here to avoid complexity. An autoencoder is set up for both horizontal and vertical patches separately. A total of 1,94,560 of both horizontal and vertical patches are extracted. Patches are collected from normal images as well as tumor images having non-tumor parts. The patches are collected manually from non-tumor parts to avoid errors in the collection. Horizontal patches of size 16 × 64 and vertical 64 × 16 are applied to the corresponding autoencoder after being normalized. The hidden layer of both autoencoder is set to a size of 64, 128, and 256 for analysis. Sparsity Regularization and L2WeightRegularization are set to default values 1 and 0.001 respectively. A sparse mean square error loss function is employed for training and the maximum epoch is set to 4000. The learning rate was found to be decreasing for each iteration in the training process. Autoencoder is implemented using the Neural Network Toolbox of MATLAB.
C. **Training and Optimization**
  The dataset consists of four set of 760 images of gliomas and meningioma were used for training and validation. While for training the network MRI with no tumors is selected. The patches are extracted from true images and also from the tumor images excluding the tumor parts. Around 1,94,560 horizontal and vertical patches are procured by this process and normalized as explained above before applied to the network architecture for training. A logistic sigmoid transfer function is used for both encoder and decoder parts respectively. SGD optimizer is used for optimization with an initial learning rate of 0.001 and the maximum epochs used are 4000. The network is trained in MATLAB 2018a with a processor capacity of 3.7 GHz with NVIDIA GPU and 16 GB of RAM. The obtained results along with a comparison with other networks are explained in the next section.
D. **Evaluation**
  Three evaluation metrics are considered to measure the performance of the proposed system such as Dice Similarity Coefficient (DSC), Positive Predictive Value (PPV), and Sensitivity. The DSC is a measure of overlap between the ground truth and the automatic segmentation. It is given by
  $$DSC = \frac{2TP}{{FP + 2TP + FN}}$$
  where TP, FP, FN are the True Positive, False Positive, and the False Negative detections respectively. PPV is measured from the TP and FP is defined as,
  $$PPV = \frac{TP}{{TP + FP}}$$
  Finally, Sensitivity measures the proportion of positives that are correctly identified, being defined as
  $$Sensitivity = \frac{TP}{{TP + FN}}$$

## Results and discussion

**Fig. 2:**
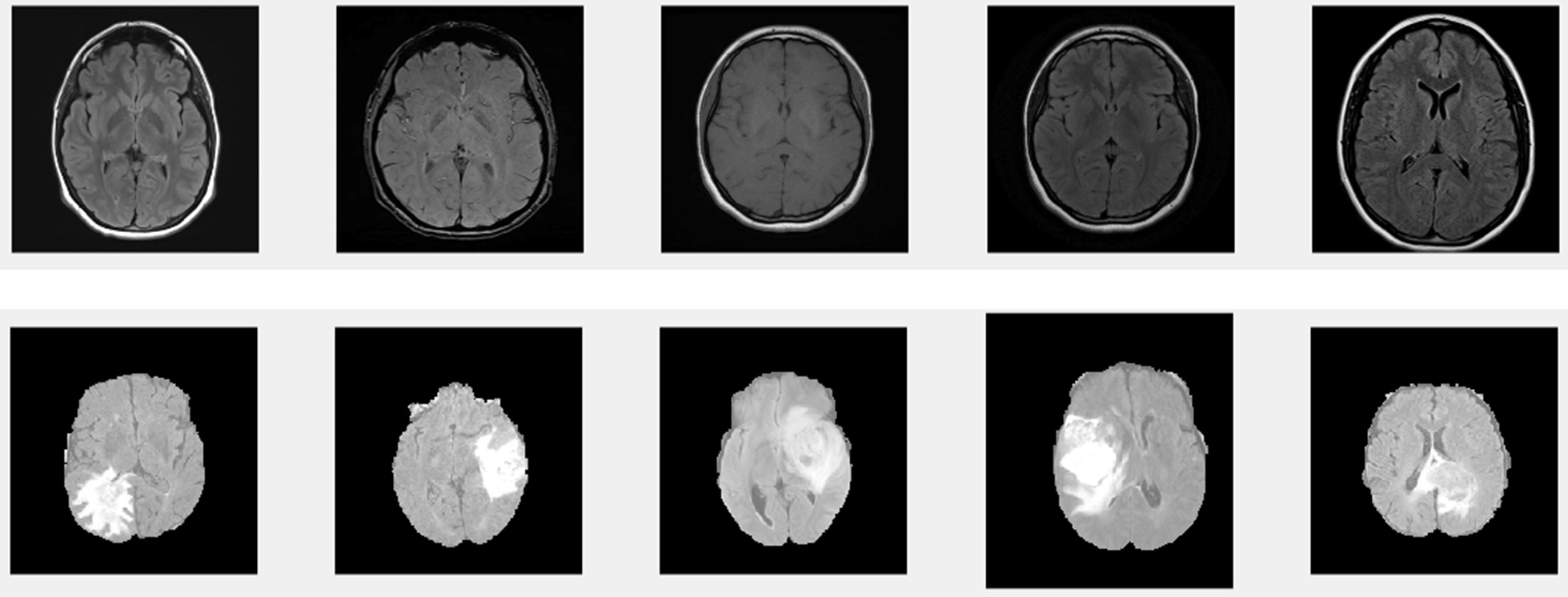
Top row: example of normal MRI images from HCP data set. Bottom row: brain tumor images from BRATS 2015 datset

Brain MRI images are having a highly complex structure. Figure 2 shows the sample images from the HCP and BRATS 2015 dataset. The top row shows the normal MRI images from the HCP dataset and the bottom row are MRI images with the tumor from the BRATS 2015 dataset. The images from the HCP dataset have the skull regions present. The first two images in the second row are meningioma variety and the rest are glioma images. We use only normal images for training. To include more data diversity we used a non-tumor part of tumor images for training. During the inference, the final decoder makes a reconstruction error for tumor pixels which is identified as anomalous samples.

**Fig. 3:**
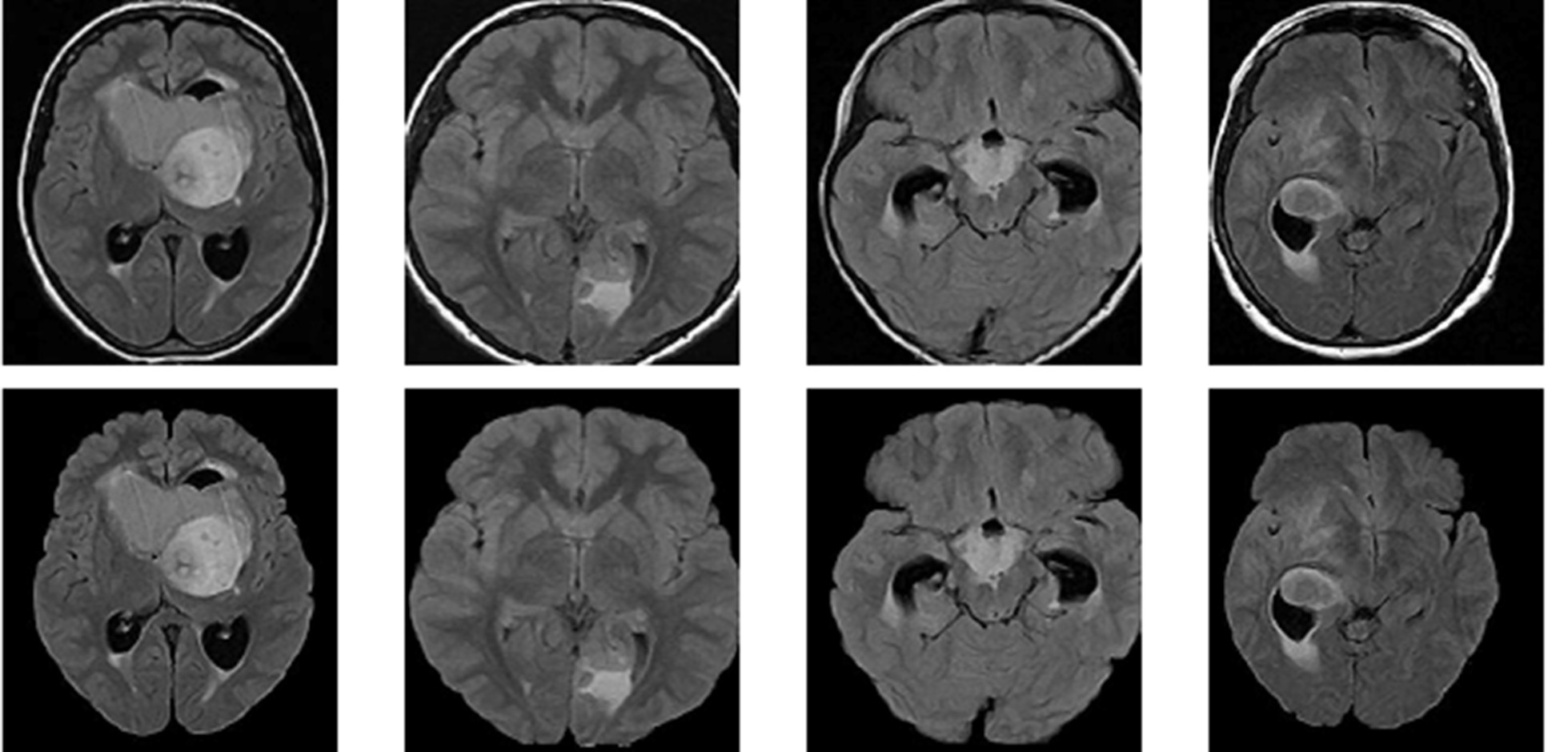
Top row: brain tumor images with Skull regions. Bottom row: skull removed images using active contour method

The first step in the process is to remove the skull part from the brain region to avoid unnecessary detection of skull part as tumor. This is a highly difficult process since the tumor can come near the edge if the skull part**.** To avoid the detection of skull regions they are stripped out before given to the tumor segmentation. An active contour algorithm proposed in [36] is used for the purpose. The idea of an active contour model is to iteratively shrunk or expand an initially closed curve with respect to the boundary of the object depending on the some parameters of the image. In [36] the authors use a two zero level curves which represent the inner and outer regions of the grey level of the cortex. Both these level set equations are driven to inner or outer boundary by a force term obtained from the intensity distribution of the MRI image. The results of the process is shown in Fig. 3.

**Fig. 4:**
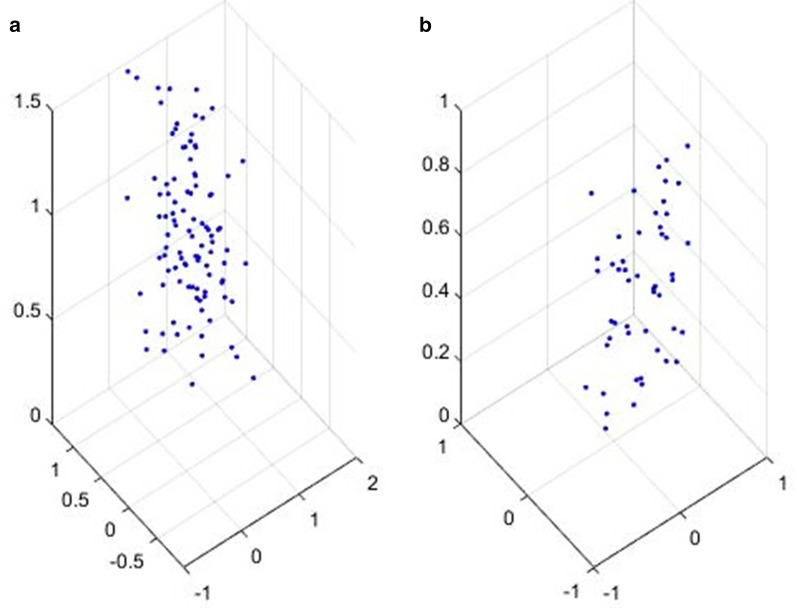
Plot **a** shows the Latent space features collected from two encoders and one auxiliary encoder. Plot **b** is the lower-dimensional features obtained after the SVD method

The latent space representation of single autoencoder and the reduced representation by SVD are shown in Fig. 4. 3D scatterplot is used to represent the latent features. Multiple loss functions are to be calculated from Dual encoder and auxiliary encoder data before giving the features to SVD for dimensionality reduction. The obtained features were statistically more independent as compared to features from the encoder outputs. The final decoder then analyses the features for the reconstruction of the samples.

**Fig. 5:**
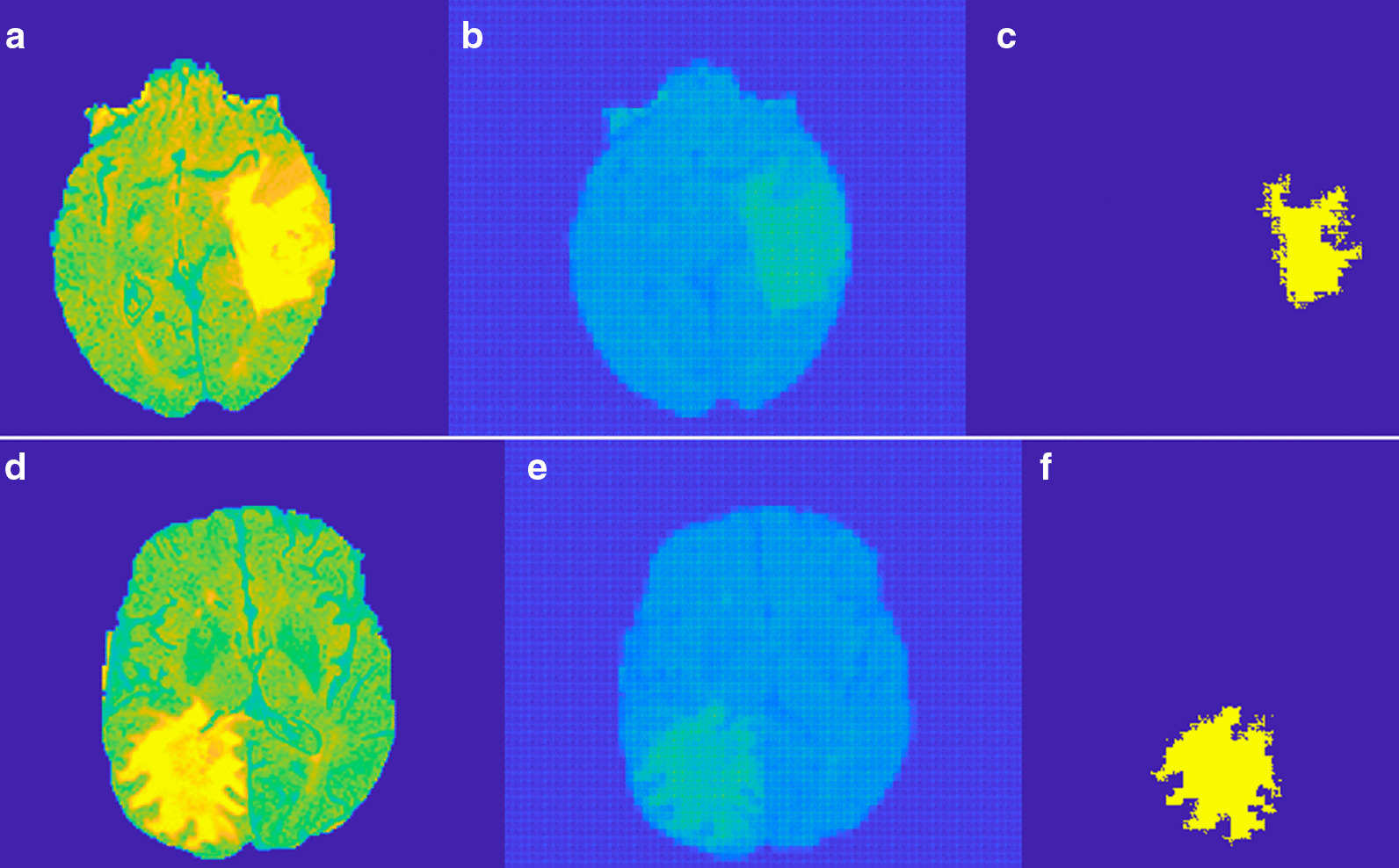
Example of the proposed method on BRAT 2015 Meningioma. **a** Original tumor image, **b** Dual autoencoder inference. **c** Segmented tumor

**Fig. 6:**
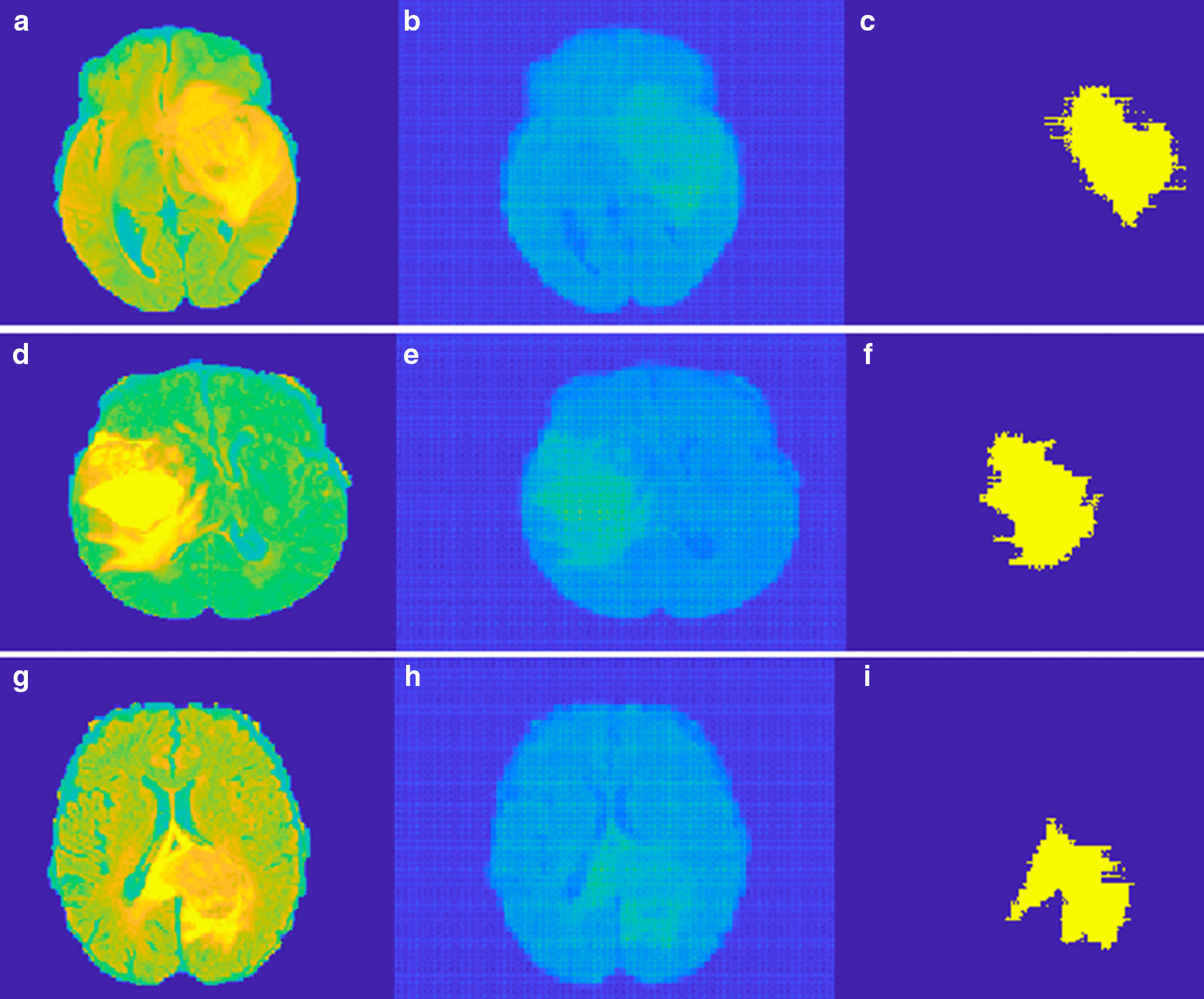
Example of the proposed method on BRAT 2015 Gliomas. **a** Original tumor image, **b** Dual autoencoder inference. **c** Segmented tumor

The idea behind the anomalous detection of tumors is that the autoencoder failed to reconstruct the tumor pixels. We tried both meningioma and gliomas tumor for testing the performance of the proposed method. We tried to detect the tumor present in the BRATS 2015 dataset as shown in the first column of Figs. 5 and 6. Figure 5 represent Meningioma images while Fig. 6 shows the Glioma tumor images. The detection performance of autoencoder is shown in the second column of both Figs. 5 and 6. Some post-processing operations for removing the unwanted parts detected as tumor pixels in Meningioma while Gliomas require further post-processing like region filling. In Glioma images tumor is not grown from a point rather it spreads randomly. This created voids in tumor parts. Region filling and connectivity operations are performed to get the final tumor segmentation. The results obtained after post-processing is shown in the third column of both Figs. 5 and 6. These results are obtained using a patch size of 16 × 64, 64 × 16 for horizontal and vertical patches respectively. Even though we tried other patch sizes the best results are obtained for a size 16 × 64 for horizontal and 64 × 16 for vertical patch.

Three evaluation metrics are employed here to measure the performance of the proposed system namely DSC, PPV, Sensitivity. The proposed method uses another patch size of 16 × 32 but with lower performance compared to a patch of 16 × 64. Also, the model is tested for a conventional square patch of size 16 × 16. The obtained results are given in Table 1. The anomalous tumor prediction in [37] uses the Area Under the Curve (AUC) for the evaluation. The paper also compares the classical autoencoder and different types of VAE for their performance in anomalous tumor detection. The comparison of these with the proposed method is given in Table 2. The performance of deep learning based CNN [27] and RescueNet [32] is slightly better compared to the proposed method in terms of the evaluation metrics. But the proposed method has the advantage of less database requirement and improved running time. The running time for training 450,000 patches in deep CNN [27] is 8 min an Intel Core i7 3.4 GHz machine with NVIDIA GeForce GTX 980 GPU. The GAN model in [32] the weight parameters of networks are updated in 200 epochs on NVIDIA DGX station with processor 2.2 GHz, IntelXeonE5-2698,NVIDIATeslaV100 4 × 16 GB GPU for a 155 slices of brain of size 240 × 240. The complex Generator design results in a running time of 10 min for the training. But for the proposed method the number of patches used is 1, 94,560 and the training time is 2 min for the same setup. Similarly the inference time of deep CNN is found to be 18 s in a test dataset of 10 images of size 512 × 512 while for the proposed method it is 10 s.

**Table 1:** Study and comparison of the proposed method and various deep learning and machine learning methods

Method	Patch size	Type	DSC	PPV	Sensitivity
Proposed	16 × 64	Meningioma	0.84	0.88	0.89
	16 × 32		0.83	0.85	0.87
	16 × 16		0.81	0.82	0.83
	16 × 64	Glioma	0.82	0.84	0.86
	16 × 32		0.81	0.825	0.85
	16 × 16		0.78	0.80	0.81
				0.85	0.86
CNN [27]	16 × 16	Glioma	0.88	0.89	0.92
SVM [5]		Glioma	0.80	0.81	0.82
KNN, SVM [18]		Various	0.81	0.815	0.83
ANN [23]		Various	0.83	0.82	0.84
RescueNet [32]		Gliomas	0.94	0.85	0.88
3D-GAN [33]		Gliomas	0.87	0.88	0.88

**Table 2:** Comparison of AUC for different autoencoder architectures

Method	AUC
Proposed	0.995
Adversarial [37]	0.994
AE	0.764
VAE	0.816
VAE-H	0.74
eeVAE	0.867
ADAE	0.892
EB	0.95

## Conclusion

Dual autoencoder based architecture is proposed here for tumor detection and latent space optimization is done using SVD. Instead of conventional square patches, we employed horizontal and vertical patches. This keeps the complex spatial information in the patches. Performance analysis shows this provides better results than square patches. The reconstruction error of the final decoder from the optimized latent features is used to identify tumor pixels from normal pixels. The experimental analysis states that the proposed method can be compared to a deep learning method in terms of performance but with less design complexity. The huge dataset requirement, complex design, and rigorous training cost of deep learning-based models are bypassed using normal brain samples for training and testing. The Dice core similarity of Deep CNN is 0.88 while the highest is for RescueNet 0.94 while the proposed method score is 0.84. For the running time the proposed method outperforms all other methods. The training time for proposed method is 2 min while that of Deep CNN and RescueNet are 8 and 10 min respectively. This is due to the low complexity in the design and reduced database requirement for the proposed work.Treating tumor pixels as anomalous samples lead to the development of unsupervised tumor segmentation models. Overlapped skull and tumor regions still create performance degradation in the proposed system. In the future, this can be overcome by using a semi-supervised model where the tumor pixels are treated as forged on normal brain images. Due to its simplicity in design, we hope the proposed method using autoencoder will lead to more sophisticated anomaly prediction designs.

## Data Availability

The data is acquired from a publically available data set https://figshare.com/articles/brain_tumor_dataset/1512427; https://www.smir.ch/BRATS/Start2015. The datasets and materials used and/or analysed during the current study available from the corresponding author on reasonable request.
